# Bioecological Aspects of Species of the Subgenus *Mansonia* (*Mansonia*) (Diptera: Culicidae) Prior to the Installation of Hydroelectric Dams on the Madeira River, Rondônia State, Brazil

**DOI:** 10.3390/tropicalmed8100479

**Published:** 2023-10-22

**Authors:** Francisco Augusto da Silva Ferreira, Fábio Medeiros da Costa, Ayrton Sena Gouveia, Rosemary Aparecida Roque, Wanderli Pedro Tadei, Vera Margarete Scarpassa

**Affiliations:** 1 Programa de Pós-Graduação em Entomologia, PPGEnt, Instituto Nacional de Pesquisas da Amazônia, INPA, Manaus 69067-375, Brazil; fabiologocosta@gmail.com; 2 Programa de Pós-Graduação em Biologia Parasitária, Instituto Oswaldo Cruz, Rio de Janeiro 21041-250, Brazil; ayrton.gouveia@fiocruz.br; 3 Laboratório de Malária e Dengue, Coordenação de Sociedade, Ambiente e Saúde COSAS, Instituto Nacional de Pesquisas da Amazônia, INPA, Manaus 69067-375, Brazil; rosemary.roque@inpa.gov.br (R.A.R.); 4 Laboratório de Genética de Populações e Evolução de Mosquitos Vetores, Coordenação de Biodiversidade, COBIO, Instituto Nacional de Pesquisas da Amazônia, INPA, Manaus 69067-375, Brazil; vera@inpa.gov.br

**Keywords:** Mansoniini, Amazonian region, *Mansonia titillans*, Culicidae

## Abstract

The objective of this study was to evaluate ecological aspects of *Mansonia* species before the construction of hydroelectric plants on the Madeira River, and thus enable the assessment of the impact of these projects on mosquitoes. A total of 199 samplings were carried out between November 2003 and August 2004, using the technique of attraction with protection. Temporal distribution was evaluated from monthly incidence values obtained from the bite index per man/hour. Relative abundance was subsequently calculated to evaluate the spatial distribution of species, according to land use and municipal districts; furthermore, the pattern of hematophagous activity was evaluated from 12-h and 4-h samplings. The data were analyzed according to the negative binomial distribution and generalized linear models to estimate the influence of environmental factors on the presence and abundance of *Mansonia*. A total of 1479 specimens were collected, distributed among four species—*Mansonia titillans* (87%), *Mansonia humeralis* (6.3%), *Mansonia amazonensis* (6%), and *Mansonia indubitans* (0.5%), and spatial distribution analysis showed *Ma. titillans* to be dominant. Hematophagous activity had peaks between 6:00 p.m. and 8:00 p.m. and species incidence was higher during the rainy season and in areas where domestic animals are raised. Therefore, the region studied presented characteristics favorable to the reproduction of *Mansonia* even before the construction of the hydroelectric plants and after construction, these conditions were enhanced, due to the increase in the availability of breeding sites for immatures and blood sources for females, as a consequence of changes in the environment.

## 1. Introduction

Mosquitoes (Culicidae) transmit the pathogens that cause malaria, yellow fever, dengue, Zika, and chikungunya, among others, resulting in a high number of human deaths and hospitalizations in different regions of the world [1]. The mosquito genus *Mansonia* of the subfamily Culicinae has two subgenera: *Mansonioides* Theobald, 1907 consisting of ten oriental and two Ethiopian species, and *Mansonia* Blanchard, 1901 consisting of fifteen Neotropical species [2,3,4,5].

At least twelve species of *Mansonia* have been recorded in Brazil, of which six are present in the Amazon region—*Mansonia amazonensis* (Theobald), *Mansonia flaveola* (Coquillett), *Mansonia humeralis* Dyar & Knab, *Mansonia indubitans* Dyar & Shannon, *Mansonia pseudotitillans* (Theobald), and *Mansonia titillans* (Walker) [3,4,5,6,7]—although molecular studies suggest that the diversity is greater [8,9]. Species of *Mansonia* are often found in forest environments, but they can also occur in urbanized areas [10]. Females are aggressive and voracious during hematophagy, with preferential nocturnal activity [10,11].

The epidemiological importance of *Mansonia* is related to the participation of some species in the transmission cycle of arboviruses and filariasis [12,13,14,15,16,17,18,19]. Furthermore, a new virus of the family Tymoviridae was recently isolated from *Mansonia* specimens collected in the city of Porto Velho; nonetheless, few studies have assessed the medical importance of these mosquitoes in Brazil [20]. Recently, De Sousa et al. (2023) [21] carried out an investigative study to evaluate the distribution of arboviruses in *Mansonia humeralis* and reported 34 positive pools for the Mayaro virus (MAYV), obtained from females captured in the district of Jaci Paraná, municipality of Porto Velho, in the region known as the Madeira River hydroelectric complex.

Uncontrolled reproduction of species of *Mansonia* is generally associated with the imbalance of aquatic flora in areas influenced by hydroelectric projects [5,20]. Macrophytes serve as reproduction sites in these areas, as they provide oxygen for the respiration of larvae and pupae [22,23,24]. This sequence of phenomena occurred in the area influenced by the Tucuruí hydroelectric plant in the state of Pará, with a marked population growth of species of *Mansonia* during the post-construction phase, when each collector captured 600 mosquitoes per hour [11].

In the Amazon, the presence of lakes with macrophytes in floodplain areas, associated with changes in land use and with the raising of domestic animals on farms, farmstead, and rural villages, contributes to the proliferation of *Mansonia* species. This is because there is an increase in the availability of blood sources for females, favoring the reproduction of these mosquitoes in the environment. Some species that are generalists in capturing blood for food, such as *Ma. titillans* and *Ma. humeralis*, benefit from these landscapes, as they consume the blood of domesticable animals such as cattle, horses, goats, birds, and also humans [25]. These interrelations are present in the areas covered by hydroelectric plants and are important for understanding the spatial distribution and the dispersion capacity of species in the environment [26].

The record of the relationship between *Mansonia* species and areas of influence of hydroelectric projects, first observed in Tucuruí, boosted several studies in the region of the so-called Madeira River hydroelectric complex, in order to understand different aspects related to taxonomy, vector competence of the species, seasonality, characteristic breeding grounds for immature forms, and dispersal patterns of *Mansonia* species, mostly carried out in the post-construction phase of the hydroelectric dams [8,9,20,21,22,26,27,28]. In this study, we approach pioneering data from the pre-construction phase of the Jirau and Santo Antonio hydroelectric plants, evaluating ecological aspects, space–time distribution, patterns of hematophagous activity, and the relationships of the species with the characteristics of the local landscape. This information can be used to design efficient population control measures and evaluate possible impacts generated from the construction of hydroelectric plants in the region. The present study, therefore, aimed to evaluate bioecological aspects of species of the subgenus *Mansonia* (*Mansonia*) in the region of the upper Madeira River, before the construction of the Jirau and Santo Antônio hydroelectric power plants.

## 2. Material and Methods

### 2.1. Study Area

**Figure 1:**
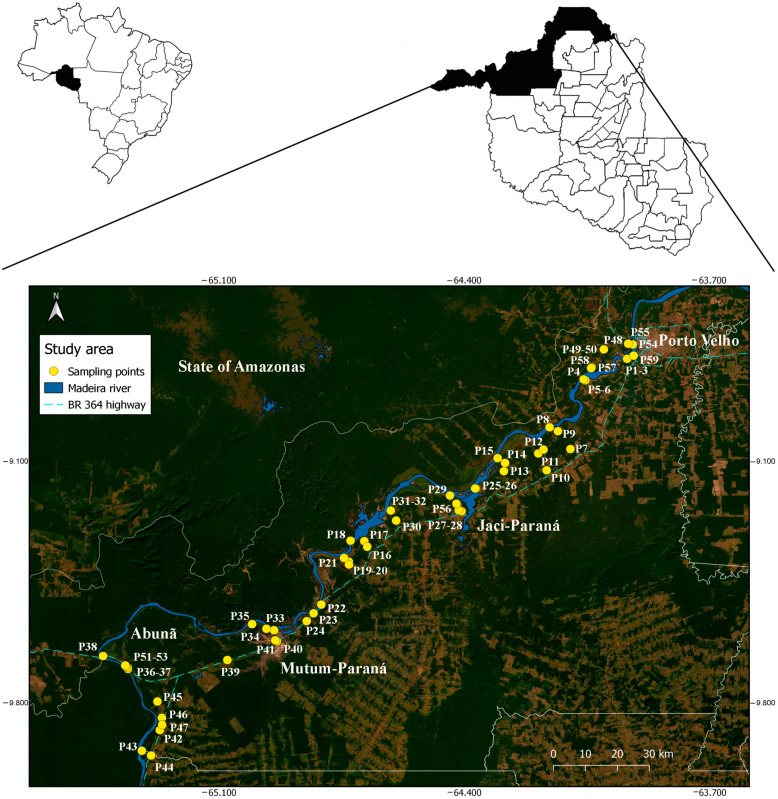
Mosquito sampling points in the upper Madeira River region, Porto Velho, Rondonia, Brazil.

The study took place in the state of Rondonia, which occupies 23.7 million hectares, representing 4.7% of the Legal Amazon. The climate is classified as Aw [29], tropical rainy, with an average annual temperature of 25 °C (20.7–31.5 °C), a relatively dry season and a rainy season, with marked rains between September and May, for a total annual rainfall of above 2000 mm [30]. The study was carried out in the districts of Porto Velho (capital), Jaci Paraná, Mutum Paraná, and Abunã, located in northern Rondônia. The population size of municipality in 2019 was estimated at 52,9544 inhabitants [31]. The districts of Porto Velho (capital) and Jaci Paraná are located in the area of influence of the Santo Antônio hydroelectric plant, while the districts of Mutum Paraná and Abunã are located in that of the Jirau hydroelectric plant (Figure 1). The district of Mutum Paraná was relocated after the filling of the reservoir, which took place in 2013, forming the village of Nova Mutum Paraná, located further downstream.

### 2.2. Mosquito Sampling

**Figure 2:**
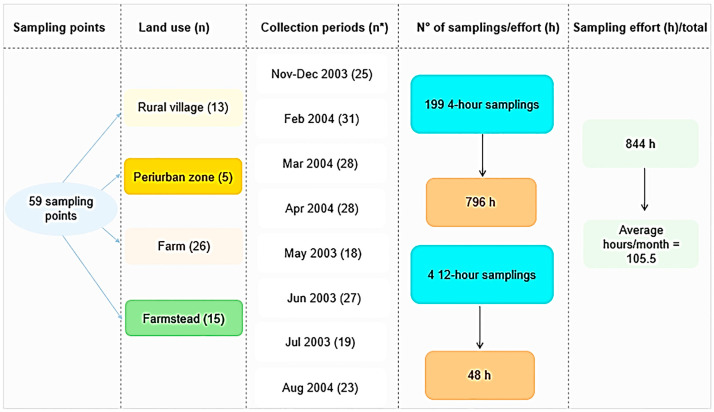
Sampling design used to evaluate the bioecological aspects of *Mansonia* species, in the upper Madeira River region, Porto Velho, Rondonia, Brazil (* number of nights).

A total of 199 samplings were carried out at 59 points, according to land use, namely: Farm—places with cattle ranching (26 points); Periurban zone (5 points); Farmstead—places without cattle ranching (15 points); and Rural village (13 points). Sampling in peridomiciliary environments took place during monthly campaigns, carried out as follows: 1—November–December (2003); 2 to 8—February to August (2004)—totaling eight collection periods; most sampling points were located on the right bank of the Madeira River, where there is great anthropic pressure and several dwellings (Figure 2). For this, six field collectors were needed, performing six points visited per night. The collectors did not have a fixed point for sampling, that is, they were randomly distributed throughout the area; each collector remained at the sampling point for four consecutive hours (6:00 p.m. to 10:00 p.m.), and four 12-h collections were carried out, two in the rainy season and two in the dry season (6:00 p.m. to 6:00 a.m.). Differentiated times were established to evaluate the hematophagic activity patterns of *Mansonia* species.

Adult specimens were acquired using the human landing catches (HLC) with protection and electronic entomological aspirators for capture (INPA—Ethics Committee—Opinion N° 3.474.088). Mosquitoes attracted by human odor (kairomone) were aspirated and transferred to paraffin cups with the help of a Castro capturer. The team involved in the sampling wore personal protective equipment that consisted of black socks, long denim pants, long-sleeved shirts, closed shoes, and a hat. Sampled mosquitoes were transported to the Laboratório de Malária e Dengue, of the Instituto Nacional de Pesquisas da Amazônia—INPA, and identified using the key of Forattini (2002) [32] and the descriptions of Barbosa (2007) [3]. Specimens were preserved following Belkin (1967) [33] and Forattini (1962) [34] and deposited in the INPA Invertebrate Collection.

### 2.3. Data Analysis

Monthly relative abundances of species of *Mansonia* were calculated to determine the species composition of the studied area. The temporal distribution of species was evaluated by calculating the bite rate per man/hour, obtained from the ratio between the number of mosquitoes collected (Nm), number of collectors (NC), and number of collection hours (NH) [35]. Sampling point data were used to evaluate the spatial distribution of the species according to district (Porto Velho, Jaci Paraná, Mutum Paraná and Abunã), in the area of the Madeira River. The pattern of hematophagous activity of species of *Mansonia* was evaluated considering collections of 12 (6:00 p.m. to 6:00 a.m.) and 4 (6:00 p.m. to 10:00 p.m.) consecutive hours. These hourly collections were essential to know the peaks of greater hematophagic activity, as the mosquitoes were quantified according to the time of capture. The information was recorded in field spreadsheets.

The sampling points were properly characterized according to the distance from the water (m) and the forest (m), number of residents, land use, and animal husbandry (goat, horse, cat, dog, chicken, and duck); this information was completed in field worksheets and subsequently inserted into the database. Subsequently, the data were subjected to negative binomial distribution analysis to assess the presence/absence of *Mansonia* mosquitoes based on the following predictors—distance from water (m), distance from forest (m), number of residents, land use, and livestock of animals. Initially, the data were analyzed using the Pascal distribution, through the negative binomial model, aiming to evaluate the influence of the predictors on the presence of *Mansonia* species. Afterwards, the data were submitted to analysis of Generalized Linear Models, with a Poisson distribution model, to evaluate the influence of the predictors on the abundance of *Mansonia*. The analyses were performed with the aid of the R software (version 4.0.2), “glm” function of the “gamlss” package.

## 3. Results

A total of 1479 specimens distributed in four species of *Mansonia* were collected: *Ma. amazonensis*, *Ma. humeralis*, *Ma. indubitans* and *Ma. titillans*. The most abundant species was *Ma. titillans* (86.6%), followed by *Ma. humeralis* (6.5%), *Ma. amazonensis* (6.2%), and *Ma. indubitans* (0.5%). There were specimens that could not be identified at a specific level, which were denominated as *Mansonia* spp. (0.2%). According to bite rate per man/hour, the incidence of *Ma. indubitans* ranged from 0 to 0.012, being highest in February, while that of *Ma. amazonensis* ranged from 0.006 to 0.1, being highest in June. The incidence of *Ma. humeralis* ranged from 0.025 to 0.055, while that of *Ma. titillans* ranged from 0.24 to 1, both being highest in July (Table 1). In the month of August, no specimens of *Mansonia* were captured.

**Figure 3:**
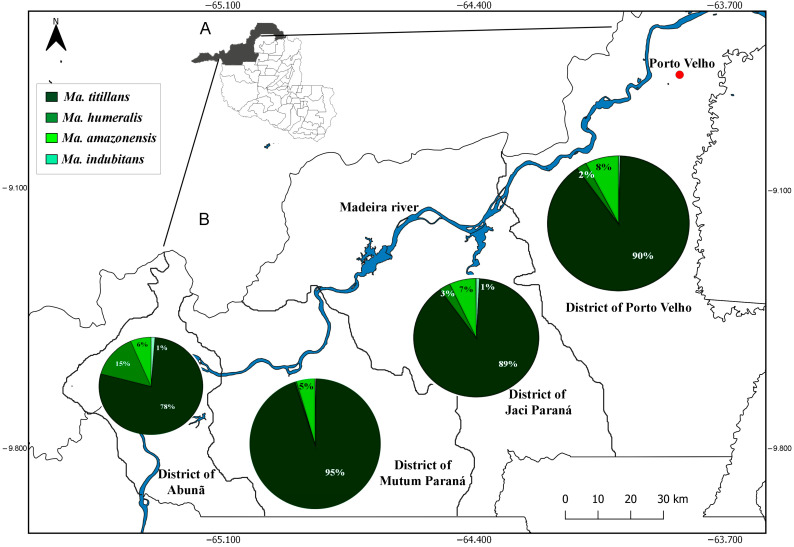
Spatial distribution of species of *Mansonia* in the upper Madeira River region (A), according to the municipal districts of Porto Velho (B), Rondonia, Brazil.

The spatial distribution data show that *Ma. titillans* was widely dispersed among the four districts analyzed (Porto Velho 90%, Jaci Paraná 89%, Mutum Paraná 95%, and Abunã 78%), and present in the periurban area of the city of Porto Velho. On the other hand, *Ma. humeralis* occurred at low abundance in the periurban region (2%) but with increased abundance in the most distant points of the city (Abunã 15%). *Mansonia amazonensis* had higher abundance near the urban perimeter of Porto Velho, and lower in the other districts, while *Ma. indubitans* had low abundance in all the analyzed districts (Figure 3).

**Figure 4:**
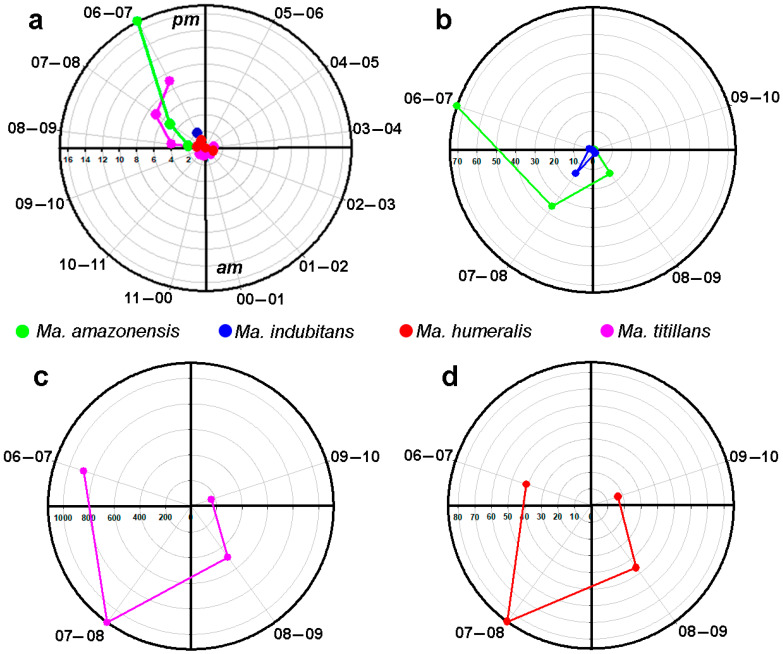
Hematophagous activity of species of *Mansonia* collected in the upper Madeira River region, Porto Velho, Rondonia, Brazil, (**a**) 12 h of collection; (**b**–**d**) 4 h of collection.

The 12-h peridomicile sampling revealed that *Ma. titillans* had higher activity between 6:00 p.m. and 7:00 p.m., with a decrease in later hours. On the other hand, hematophagous activity of this species was observed in the interval between 3:00 and 4:00 a.m. The observed behavior for *Ma. amazonensis* was similar to that of *Ma. titillans*, with greater hematophagous activity between 6:00 and 7:00 p.m., but not extending beyond 8:00 to 9:00 p.m. *Mansonia humeralis* also showed hematophagous activity between 6:00 and 7:00 p.m., extending to the interval 2:00 to 3:00 a.m. Hematophagous activity of *Ma. indubitans* was only recorded between 6:00 and 7:00 p.m. (Figure 4a). The four-hour sampling revealed a peak of hematophagous activity for *Ma. amazonensis* between 6:00 and 7:00 p.m., with a decrease in later intervals. In contrast, peak hematophagous activity for *Ma. titillans* and *Ma. humeralis* was between 7:00 and 8:00 p.m., with a decrease in subsequent intervals. *Mansonia indubitans* had low hematophagous activity in all temporal intervals (Figure 4b–d).

**Figure 5:**
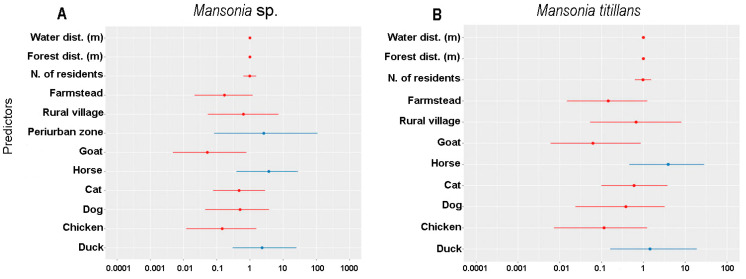
Pascal distribution analysis, using a negative binomial model to evaluate the influence of predictors on the presence of *Mansonia* sp. (**A**) and *Mansonia titillans* (**B**), in the upper Madeira River region, Porto Velho, Rondonia, Brazil.

Pascal’s distribution analysis indicated that among the analyzed predictors, the periurban area, the presence of horses and ducks were considered important for the presence of *Mansonia* mosquitoes (Figure 5A). On the other hand, *Ma. titillans* was present mainly in those places where horses and ducks were maintained or raised (Figure 5B).

In the analysis of generalized linear models—GLM, two models were obtained that best explained the relationship between *Mansonia* and the analyzed predictors. The first was performed confronting predictors versus *Mansonia* spp. and the other with predictors versus *Ma. titillans*. We can observe that the first model demonstrates that there was a significant influence of the type of land use—farmstead (*p* = 0.027) and the presence of goat (*p* = 0.021) and chicken (*p* = 0.032)—on the abundance of *Mansonia* spp. in the studied area. These same predictors also influenced the abundance of *Ma. titillans*—land use—farmstead (*p* = 0.024) and the presence of goat (*p* = 0.023) and chicken (*p* = 0.026) (Table 2).

## 4. Discussion

Aspects of the bioecology, taxonomy, and behavior of *Mansonia* mosquitoes remain little studied, even after their proven relationship with hydroelectric projects in Brazil, as first observed in the Amazon region for the area of influence of the Tucuruí hydroelectric plant [12]. The present study found the abundance of species to be low, when compared to that found in the area of influence of the Tucuruí hydroelectric plant, where a total of 33,458 specimens of *Mansonia* were captured in 10 sampling points.

The dominance of *Ma. titillans* and *Ma. humeralis* (93.3%) recorded here corroborates the findings of Galardo et al. (2022) [27], for controlled studies in the same area, where the two species represented 90.1% of the specimens captured between 2015 and 2019, shortly after the construction of the Santo Antonio hydroelectric plant. Thus, the dominance of these species over other species of *Mansonia* did not change between pre- and post-construction on the Madeira River (Table 1). Similar results were also reported by Scarpassa et al. (2022) [9] after the construction of the Jirau hydroelectric plant.

The predominance of *Ma. titillans* reinforces the eclectic and opportunistic behavior of this species, which uses forested areas as shelter and open areas for foraging [36,37], feeding on several species of animals including birds, rodents, humans, horses, lizards, cattle, capybaras, and frogs [38], with high occurrence around rural villages, cities, and on farms [39] (Table 1; Figure 3). Conversely, *Ma. titillans* was abundant at points in periurban areas, corroborating Navarro-Silva et al. (2004) [10], who found this species inhabiting a forest fragment in the urban area of Curitiba, state of Paraná, southern Brazil. Data obtained by Scarpassa et al. (2022) [8] studying the DNA barcode of species of *Mansonia* collected in the same area (Jirau hydroelectric plant) suggest that *Ma. titillans* represents a species complex. Therefore, the occurrence of this species in different types of environmental conditions may be due to the existence of cryptic species in the area, which is reflected in greater adaptive capacity, factors that must be considered when control measures are implemented.

Prior to the construction of the hydroelectric plants, the right bank of the Madeira River suffered intense anthropic pressure from deforestation and mining, which likely influenced the diversity of species obtained in the present study. Hutchings et al. (2008) [40] reported a total of 1802 specimens of *Mansonia* in 367 samplings carried out in 50 locations along the Solimões–Amazon River channel, representing six species—*Ma. amazonensis*, *Ma. flaveola*, *Ma. humeralis*, *Ma. indubitans*, *Ma. pseudotitillans*, and *Ma. titillans*. Of these, *Ma. amazonensis* was the most abundant, differing from the present study, which found four species, and *Ma. titillans* accounting for almost 90% of the mosquitoes collected (Table 1; Figure 3). These differences between may be explained because the authors sampled in a preserved environment (six species), whereas the present study sampled an anthropized environment, resulting in lower species diversity (four species) and greater dominance (e.g., *Ma. titillans*). Based on the results of Tadei et al. (1991) [11] and of the present study, *Ma. titillans* seems to benefit from anthropized environments, whereas *Ma. amazonensis* may benefit from more preserved environments.

Species of *Mansonia* showed higher densities in February, March, and April, corresponding to the rainy season, except for *Ma. indubitans*, which had low incidence throughout the studied period (Table 1). According to Fearnside (2014) [41], there is higher incidence of macrophytes in the upper Madeira River during the high-water period, when these plant groups are present on both banks of the river, opposite to what was observed in the low-water period. The macrophyte species in the region of the upper Madeira River with the highest abundance of *Mansonia* immatures were *Eichhornia crassipes* (Mart.) Solms (n = 14,438), followed by *Salvinia* sp. (n = 2292) and *Pistia stratiotes* (Jalkumbhi) (n = 1877), with a peak population of immatures during the rainy season (Gil et al. 2021) [22]. In the month of August, no specimens of *Mansonia* were collected, due to the dry period that reduces the number of available breeding sites, and several fires with large amounts of smoke, these factors may have negatively influenced the density of *Mansonia*.

The 4-h and 12-h samplings revealed higher hematophagous activity of *Mansonia* females between 6:00 and 8:00 p.m. (Figure 4a–d). These findings differ from those of Cruz et al. (2009) [42], who observed greater activity of mosquitoes of the subfamily Culicinae between 8:00 p.m. and 9:00 p.m., in peridomicile areas located in the region of the Madeira River hydroelectric complex. This may have occurred because the authors included *Culex*, *Coquillettidia*, and *Psorophora* specimens in the analyses, resulting in variations in patterns of hematophagic activity, since biting activity varies within the Culicinae subfamily. On the other hand, the biting activity data recorded in the present study corroborate Galardo et al. (2022) [27], who also observed peaks of hematophagous activity between 7:00 and 8:00 p.m. for *Ma. titillans* and *Ma. humeralis* in the same region. 

The biting activity patterns of culicids show great interspecific and even intraspecific variation, as observed by Zimmerman et al. (2013) [43], studying the nocturnal cycles of malaria vector bites in Brazil. Still in this sense, Charlwood (1996) [44] suggests that the age of the population, the phase of the moon and the distance from the oviposition or mating site can influence the patterns of hematophagic activity.

The studied predictors responded differently for the variables of presence/absence and abundance of *Mansonia* species (Table 2; Figure 5). The presence of *Mansonia* spp. was associated with duck and horse rearing in the vicinity of the peri-urban area and the presence of *Ma. titillans* was also associated with the rearing of these animals. According to the data obtained by Alonso et al. (2023) [25], carried out in the same region, the hematophagous behavior of *Mansonia* species was heterogeneous, where *Ma. amazonensis* showed high affinity for *Bos taurus* Linnaeus, *Ma. humeralis* for *Cannis familiaris* Linnaeus and *B. taurus*, in addition to *Homo sapiens* and *Equus caballus* Linnaeus, while *Ma. titillans* was highly opportunistic, feeding on the blood of *Gallus gallus* Linnaeus and *Homo sapiens* Linnaeus.

The analysis of generalized linear models showed that the abundance of *Mansonia* spp. and *Ma. titillans* were influenced by the site environment, with goat and chicken rearing. These data indicate the presence of *Mansonia* in anthropized environments, adapting to feed on different blood sources and favoring the dispersal process of the species, especially if these locations are close to lakes with macrophytes, oviposition sites for this group of mosquitoes. According to De Mello and Alencar (2021) [26], they tend to remain close to their breeding sites, but distances of up to 2000 m from the release point were recorded for *Ma. amazonensis* and *Ma. humeralis*.

The results observed in this study, carried out before the construction of the hydroelectric plants, indicated that the density of *Mansonia* species increased after construction. The studies conducted by Galardo et al. (2022) [27] in the area of influence of the Santo Antônio hydroelectric plant, also located in the same region, recorded 96,766 specimens captured over a period of five years, between 2015 and 2019, carrying out three annual collections, with a total of 15 collection periods, while the results presented here between 2003 and 2004 recorded 1479 specimens, at the end of eight collection periods. The collection effort of the authors mentioned above was 192 h/month while in this study it was 105.5 h/month (Figure 2). The transformations that occurred in the region with the expansion of floodable areas made the environment favorable for the reproduction of macrophytes, positively reflecting on the increase in the population density of *Mansonia* species.

It should be noted that there is currently no official population control program for *Mansonia* species in Brazil. Currently, some initiatives have been adopted by the private sector to expand studies on these mosquitoes, but there is still no consensus on the best strategies that can be implemented to reduce the population. Challenges include the difficulty in identifying the species, especially immature forms, and the extensive breeding sites, making it difficult to mechanically remove macrophytes or even apply larvicides. Therefore, we recommend the installation of mosquito nets on doors and windows and indoor spraying in homes close to breeding sites, constituted by farms, especially during the rainy season to reduce the successive bites from female *Mansonia*.

In addition to mosquito population control techniques, it is important to consider measures to reduce the biomass of macrophytes in the area of influence of the hydroelectric plant. There is no consensus on the best technique to be used; however, it is known that the use of herbicides poses a risk to the aquatic environment, as does the introduction of species that act in biological control. Therefore, it is necessary to strengthen ecological knowledge of macrophyte species, as each aquatic ecosystem has unique characteristics with distinct limnological properties. This will make it possible to carry out modeling studies to predict the proliferation potential of macrophyte species with a view to controlling and consequently reducing the development habitats of immature of *Mansonia*.

## 5. Conclusions

The findings of this study indicated that *Mansonia* species showed a high incidence in the rainy season, with peaks of twilight hematophagic activity. In addition, *Ma. titillans* was observed to be predominant species in the region, demonstrating highly adaptive plasticity in these environments, occupying niches near the urban perimeter as well as more distant places. The presence and abundance of *Mansonia* mosquitoes was strongly influenced by human activities and transformation of the landscape on the banks of the Madeira River, involving land use and animal husbandry, consequently increasing the sources of blood meal for these species in this environment. In this study, the density of *Mansonia* species was influenced by the flood and ebb pulses of the Madeira River and the *Ma. titillans* population responds positively to environmental changes related to land use. Furthermore, the low density found in the pre-construction period contrasts with studies carried out in the same region after the construction of the hydroelectric plants, indicating that the environmental transformations that occurred benefited the *Mansonia* mosquito population, despite not altering the composition of the species.

## Figures and Tables

**Table 1:** Bite rate per man/hour and abundance (n) of species of *Mansonia* in the region of the upper Madeira River, prior to the construction of the Santo Antônio and Jirau hydroelectric plants, in Porto Velho, Rondônia, Brazil. RA = relative abundance.

Species	Nov–Dec	Feb	Mar	Apr	May	Jun	Jul	Total	RA (%)
*Ma. indubitans*	0 (0)	0.012 (3)	0.003 (3)	0 (0)	0 (0)	0.005 (2)	0 (0)	8	0.5
*Ma. titillans*	0.34 (142)	0.68 (239)	0.45 (275)	0.5 (226)	0.85 (135)	0.24 (85)	1 (179)	1281	86.6
*Ma. amazonensis*	0.012 (9)	0.011(8)	0.006 (5)	0.013 (5)	0.009 (6)	0.1 (58)	0.007 (1)	92	6.2
*Ma. humeralis*	0.036 (17)	0.051 (29)	0.025 (8)	0.032 (10)	0.031 (1)	0.033 (13)	0.055 (17)	95	6.5
*Mansonia* sp.	0 (0)	0 (0)	0 (0)	0.003 (3)	0 (0)	0 (0)	0 (0)	3	0.2
Total	168	279	291	244	142	158	197	1.479	100

**Table 2:** Influence of environmental predictors on the abundance of *Mansonia* spp. and *Mansonia titillans* in the Upper Rio Madeira region, Porto Velho, Rondonia, Brazil.

Predictors	*Mansonia*			*Ma. titillans*		
	Incidence Rate Ratios	CI	*p*	Incidence Rate Ratios	CI	*p*
(Intercept)	2.83	0.17–236.82	0.441	102.81	4.83–10,204.69	*p* < 0.001
Distance—water (m)	1.00	1.00–1.00	0.290	1.00	1.00–1.00	0.338
Distance—forest (m)	1.00	1.00–1.00	0.282	1.00	1.00–1.00	0.308
Number of residents	0.93	0.60–1.45	0.612	0.98	0.62–1.54	0.868
Land use—Farmstead	0.15	0.02–1.14	0.027	0.14	0.01–1.24	0.024
Land use—Rural Village	0.70	0.06–8.54	0.708	0.67	0.05–8.08	0.667
Goat	0.06	0.01–0.85	0.021	0.06	0.01–0.86	0.023
Horse	4.24	0.48–32.06	0.062	3.92	0.46–28.39	0.074
Cat	0.64	0.11–4.07	0.558	0.60	0.10–3.77	0.489
Dog	0.33	0.02–2.70	0.305	0.38	0.02–3.23	0.369
Chicken	0.12	0.01–1.53	0.032	0.11	0.01–1.23	0.026
Ducks	1.42	0.16–19.44	0.741	1.44	0.16–19.04	0.727
Observations—2.160				Observations—45		
R^2^ Nagelkerke—0.175				R^2^ Nagelkerke—0.414		

## Data Availability

The data analyzed in this study can be accessed in more detail at https://repositorio.inpa.gov.br/handle/1/38744 (accessed on 15 August 2023).
